# Attractor Ranked Radial Basis Function Network: A Nonparametric Forecasting Approach for Chaotic Dynamic Systems

**DOI:** 10.1038/s41598-020-60606-1

**Published:** 2020-03-02

**Authors:** Maryam Masnadi-Shirazi, Shankar Subramaniam

**Affiliations:** 1 University of California San Diego, Department of Bioengineering, La Jolla, CA 92093 USA; 2 University of California San Diego, Departments of Bioengineering and Computer Science & Engineering, La Jolla, CA 92093 USA

**Keywords:** Applied mathematics, Computer science

## Abstract

**The curse of dimensionality has long been a hurdle in the analysis of complex data in areas such as computational biology, ecology and econometrics. In this work, we present a forecasting algorithm that exploits the dimensionality of data in a nonparametric autoregressive framework. The main idea is that the dynamics of a chaotic dynamical system consisting of multiple time-series can be reconstructed using a combination of different variables. This nonlinear autoregressive algorithm uses multivariate attractors reconstructed as the inputs of a neural network to predict the future. We show that our approach, attractor ranked radial basis function network (AR-RBFN) provides a better forecast than that obtained using other model-free approaches as well as univariate and multivariate autoregressive models using radial basis function networks. We demonstrate this for simulated ecosystem models and a mesocosm experiment. By taking advantage of dimensionality, we show that AR-RBFN overcomes the shortcomings of noisy and short time-series data**.

## Introduction

In recent years, the availability of large time-course datasets in multiple disciplines, including biology, ecology and finance has brought forth the problem of handling such data for scientific analysis^1–3^. In many studies, generalized linear models and vector autoregressive models are used for structural estimation and inference, where such systems exhibit nonlinear dynamics with time-lags, reciprocal feedback loops and unpredictable surprises^4,5^. On the other hand, equation-based models such as difference and differential equations may be used to analyze the evolution of a dynamic system, but often require some degree of prior knowledge about the nature of interactions among various system components^6^; even if the model structure is known, dimensionality poses a challenge on accurate parameter estimation of variables^7^. Furthermore, prior work has established that ecological and biological models are often ineffective in predicting the future due to the highly nonlinear nature of component interactions^8,9^.

An alternative equation-free approach suitable for non-equilibrium dynamics (including chaos) and nonlinearity is state space reconstruction (SSR) which is a model-free approach in the sense that there is no analytic formula assumption, thus allowing substantial flexibility in the nonlinearity of the system^10,11^. SSR uses lagged coordinate embeddings to reconstruct attractors that map the time-series evolution from time domain into state space trajectories. Reservoir computing is also another model-free approach for short-term prediction of chaotic dynamic systems from time-series data^12–16^.

In a notable theorem, Takens proved that the overall behavior of a chaotic dynamic system can be reconstructed from lags of a single variable^17^. Takens’ theorem was later generalized and it was demonstrated that the information from a combination of multiple time-series (and their lags) can be used in an attractor reconstruction to provide a more mechanistic model^18,19^. Nonetheless, since attractor reconstruction relies only on experimental data, the limitations of short or noisy time-series restricts the ability to infer system dynamics as a whole. Namely, SSR from short time-series provide a scarce view of a system’s mechanism, diminishing reliability of inferences. In addition, when time-series data is corrupted with observational noise, data may become meaningless and irrelevant in providing useful information for predictability. Ye *et al*. (2016) introduced an analytical approach, multiview embedding (MVE), which is based on simplex-projection’s search for nearest neighbors to perform forecasting^20^.

In this work, we treat prediction of the dynamical system as an inverse problem that involves interpolation and approximating an unknown function from a time-series data and introduce an attractor ranked radial basis function network (AR-RBFN)-based autoregressive model. Here, we use SSR to construct attractors from combinations of variables and their time-lags. Each manifold comprises information that provides unique predictive intelligence. We then assess the reconstructed manifolds’ prediction ability and rank them according to their forecast skill. By merging the top manifolds and the information contained in them, AR-RBFN is capable of recovering the dynamics of the system in a manner that outperforms model-free approaches such as MVE and nonlinear univariate and multivariate autoregressive models.

## Methods

**Figure 1:**
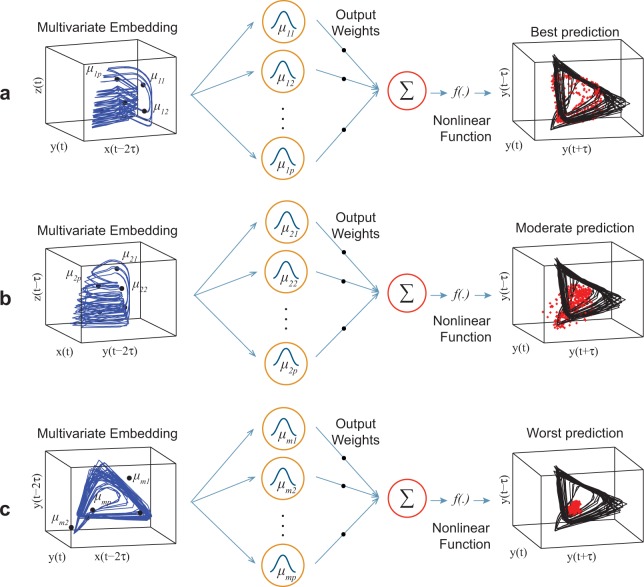
Schematic showing forecast skill of multivariate embeddings in the three-species food chain model. **(a)** Multivariate embedding reconstructed from $z(t)$, $y(t)$ and $x(t-2\tau )$ in 3-dimensional space provides the best prediction of variable $y$ using Gaussian radial basis functions with centers {${\mu }_{11},\,\ldots ,\,{\mu }_{1p}\}$. **(b)** Multivariate embedding reconstructed from $z(t-\tau )$, $x(t)$ and $y(t-2\tau )$ in 3-dimensional space provides moderate prediction of variable $y$ using Gaussian radial basis functions with centers $\{{\mu }_{21},\,\ldots ,\,{\mu }_{2p}\}$. **(c)** Multivariate embedding reconstructed from $y(t-2\tau )$, $y(t)$ and $x(t-2\tau )$ in 3-dimensional space provides the worst prediction of variable $y$ using Gaussian radial basis functions with centers $\{{\mu }_{m1},\,\ldots ,\,{\mu }_{mp}\}$.

AR-RBFN utilizes radial basis function networks (RBFN) initially proposed to perform accurate interpolation of data points in a multidimensional space^21^. Suppose we are interested in forecasting variable *y* in a three-species food chain with components, *x*, *y*, and *z*. By constructing the attractors from a combination of variables of the three-species food chain, one can look into the forecast skill of each multivariate manifold (Fig. 1).

For example, the blue manifold in Fig. 1a is an embedding constructed from variables, $Z$, $y,$ and variable $X$ delayed by two time-lags. Each multivariate embedding in Fig. 1 is mapped using a Gaussian RBFN which approximates a nonlinear function that transforms the input space of past values in each manifold to the output space of future target values:

1 $${Y}_{i}(t+\tau )=\varPsi ({M}_{i}){\alpha }_{i}+{\varepsilon }_{i},\,i=1,2,\ldots m$$

$\varPsi ({M}_{i})$ is a data matrix of nonlinear Gaussian kernel functions with the inputs being points on the ${i}^{th}$ manifold ${M}_{i}$, and ${\alpha }_{i}$ is a vector of output weights that can be fixed such that the prediction error is minimized in the minimum mean squared error sense. $m$ is the number of all possible manifold reconstructions from a combination of variables and their time-lags.

The black manifolds in Fig. 1 are reconstructed from the actual future observations of variable $y$ and the red dots are the predicted values. One can rank constructed embeddings based on their prediction accuracy (mean absolute error or correlation between observations and predictions) from the best (Fig. 1a) to the worst (Fig. 1c).

### Manifold reconstruction

**Figure 2:**
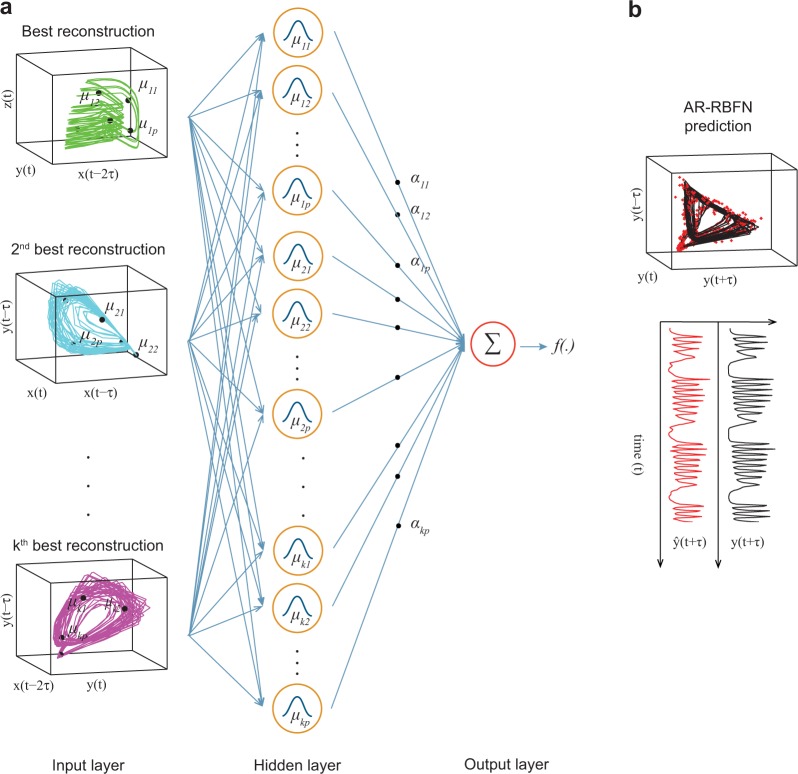
Attractor ranked radial basis function network. **(a)** Three-layer neural network takes the best $k$ predictive embeddings as its inputs. The nonlinear function *f*( · ) is estimated by fixing the $\alpha$ weights through linear optimization. **(b)** The in-sample forecast and future observations are shown by the red and black manifold (curve) in state space (time domain) respectively.

Given $N$ variables and $L$ time-lags for each variable, the possible number of manifold reconstructions in E-dimensional space grows combinatorially:

2 $$m=(\begin{array}{c}NL\\ E\end{array})-(\begin{array}{c}N(L-1)\\ E\end{array})$$

where, the first term is the number of manifolds formed by choosing $E$ of the $NL$ possible coordinates, and the second term is subtracted to account for the number of unacceptable manifolds where all $E$ coordinates are lagged; an acceptable manifold is one with at least one unlagged coordinate at the current time $t$. For example, in Fig. 1 we have reconstructed manifolds in 3-dimensional space ($E=3$) with a time lag of $0,\,\tau ,\,2\tau$ ($L=3$), which results in 64 valid manifolds. Once all reconstructions are ordered based on their prediction skill in the in-sample portion of the data, one can identify the top $k$ manifolds ${M}_{1},\,\ldots \,{M}_{k}$ in an $E$-dimensional space that will further be used in the AR-RBFN forecast. Figure 2a shows the AR-RBFN model where the top $k$ manifolds in the prediction of variable $y$ are inputs to the three-layer neural network. Each unit in the hidden layer uses a Gaussian radial basis function with centers ${\{{\mu }_{l\rho }\}}_{\rho =1}^{p},\,l=1,\,\ldots ,\,k$ as a nonlinear activation function. The one-step forecast of $y$ through attractor ranked RBFN, and the actual one-step observations of $y$ are shown in Fig. 2b in the red and black curves respectively. Figure 2 shows an in-sample one-step ahead forecast of variable $y$; our goal is to explore AR-RBFN’s predictive capability in an out-of-sample forecast scheme which we will explore later in this manuscript.

### Attractor ranked radial basis function network

Given multivariate time-series of $N$ variables $X=\{{x}_{1}(t),\,{x}_{2}(t),\,\ldots {x}_{N}(t)\};t=1,\,\ldots ,\,T$, the nonlinear attractor ranked RBFN model maps the top $k$ manifolds such that the likelihood of the nonlinear autoregressive model is maximized:

3 $${X}_{j}(t+\tau )=\varPsi (M){\alpha }_{j}+{\varepsilon }_{j},\,j=1,\,2,\,\ldots \,N$$

where,

4 $${X}_{j}(t+\tau )={[{x}_{j}(E+\tau ){x}_{j}(E+2\tau )\ldots {x}_{j}(T)]}^{T}$$

5 $$\varPsi (M)=[\varPsi ({M}_{1})\varPsi ({M}_{2})\ldots \varPsi ({M}_{k})]$$

6 $${\alpha }_{j}={[{\alpha }_{j1}{\alpha }_{j2}\ldots {\alpha }_{jk}]}^{T}$$

7 $${\alpha }_{jl}={[{\alpha }_{jl}(1){\alpha }_{jl}(2)\ldots {\alpha }_{jl}(p)]}^{T},\,l=1,2,\ldots k$$

8 $$\begin{array}{c}{M}_{l}=[\begin{array}{cc}{M}_{l}^{1} & {M}_{l}^{2}\end{array}\,\begin{array}{cc}\cdots  & {M}_{l}^{T-E}\end{array}]=[\begin{array}{cccc}{x}_{d}(E) & {x}_{d}(E+\tau ) & \cdots  & {x}_{d}(T-\tau )\\ {x}_{e}(E-{n}_{1}\tau ) & {x}_{e}(E-{n}_{1}\tau +\tau ) & \cdots  & {x}_{e}(T-{n}_{1}\tau -\tau )\\ \vdots  & \vdots  & \ddots  & \vdots \\ {x}_{b}(E-{n}_{2}\tau ) & {x}_{b}(E-{n}_{2}\tau +\tau ) & \cdots  & {x}_{b}(T-{n}_{2}\tau -\tau )\end{array}],\\ d,e,b\in \{1,2,\ldots N\},{n}_{1},{n}_{2}\in \{0,1,2\}\end{array}$$

9 $$\varPsi ({M}_{l}^{g})=[{\psi }_{1}({M}_{l}^{g}){\psi }_{2}({M}_{l}^{g})\ldots {\psi }_{p}({M}_{l}^{g})],g=1,2,\ldots ,T-E$$

10 $${\psi }_{\rho }({M}_{l}^{g})=\exp (-{\Vert {M}_{l}^{g}-{\mu }_{l\rho }\Vert }^{2}/2{{\sigma }_{l}}^{2}),\rho =1,2,\ldots p$$

*N* is the number of variables in the chaotic system. *T* is the time-series length and $\tau$ is the time increment. The value of the ${j}^{th}$ variable at time $t$ is denoted by ${x}_{j}(t)$. Equation (3) is a nonlinear autoregressive model where $\varPsi (M)$ is an activation function of the past values of variable ${x}_{j}$ that are reflected in the set of top $k$ manifolds ${M}_{1},\,{M}_{2},\,\ldots {M}_{k}$ in Eq. (5). Learning radial basis function network requires the determination of RBF weights and centers. ${\alpha }_{j}$ in Eq. (6) is the vector of weights between the target variable ${x}_{j}$ and $\varPsi (M)$. Each reconstructed manifold has a total of $p$ centers; ${\{{\mu }_{l\rho }\}}_{\rho =1}^{p}$ is the set of $p$ centers in the space of the ${l}^{th}$ manifold ${M}_{l}$ of the top $k$ manifold reconstructions. The centers are determined by a k-means clustering procedure. We can see in Eq. (8) that the columns of ${M}_{l},l=1,\,\ldots ,\,k$, that are $E$-dimensional points in the space of ${M}_{l}$, are vectors reconstructed from a combination of variables $d,e$ and $b$ and time-lags $0,{n}_{1}\tau$ and ${n}_{2}\tau$. ${\sigma }_{l}$ is the width or radius of the Gaussian radial basis function in the space of ${M}_{l}$ which is selected as the average of the Euclidean distances between each center ${\mu }_{l\rho }$ and its nearest neighbor center ${\mu }_{l\rho \text{'}}$^22^. The nonlinear functions in Eq. (9) are activation functions that calculate the kernels with respect to each of the $p$ centers (${\mu }_{l\rho }$) in the ${M}_{l}$ manifold. ${\alpha }_{jl}(\rho )$ is the weight corresponding to the kernel function ${\psi }_{\rho }({M}_{l}^{g})$. As seen in Eq. (10), the type of the radial basis function ${\psi }_{\rho }({M}_{l}^{g})$is taken as Gaussian kernels whose inputs are $E$-dimensional vectors of a combination of variables and time-lags. Here, we use $E=3$ and thus reconstruct 3-dimensional manifolds with time lags of $0,\,\tau ,\,2\tau$ with $\tau =1$.

$\alpha$ vectors are weights that are fixed such that the prediction error is minimized, and $\varepsilon$ denotes Gaussian white noise independent of the time-series. In general, one can use least squares estimation method to adjust the $\alpha$ weights in the minimum mean squared error sense. Once the $\alpha$ vectors are estimated via least squares using the library data (training time interval) that is selected randomly from the in-sample portion of the data, they are tested on the out-of-sample test set to calculate the out-of-sample forecast.

### Out-of-sample forecasting

In order to quantitatively evaluate the one-step-ahead forecast skill of the AR-RBFN, we performed an out of sample forecast scheme on the simulated ecosystem data. We generated 3000 samples for all variables in the simulated ecosystem models and discarded the first 500 samples to exclude the transient behavior of the time-series. The last 500 samples [2501 to 3000] are kept as the out of sample test set. The data in the [2001, 2500] interval is not considered in the forecasting scheme to emphasize the robustness of AR-RBFN; we need not have knowledge of the most recent events in a chaotic system to predict the future (see Fig. S1). Radial basis function based autoregressive model is performed on each of the $m$ manifold reconstructions to rank them based on their forecast skill (correlation coefficient between the observations and predictions) in the in-sample portion of the data. For the simulated time-series data, 100 libraries are randomly chosen in the in-sample portion of the data [501 to 2000]; the starting point of each library (training time interval on the in-sample portion of data) is chosen from a uniform distribution distributed in the [501 to 2000] interval. The top $k$ manifold reconstructions are selected to perform AR-RBFN forecasting (as shown in Fig. 2). The forecast skill is then calculated by averaging the mean absolute errors and correlation coefficients between the actual future observations and one-step forecasts for the 100 randomly sampled libraries. The libraries are selected in various lengths of 25, 50, 75 and 100 samples.

Due to the limited length of the mesocosm data, we used a pseudo out-of-sample forecast scheme to evaluate the forecast performance of the AR-RBFN and MVE approaches; the first 3/4 of the time-series was used as the training set, and the last 1/4 portion of the data was used as the test set. This forecast scheme is also known as the method of time-series cross-validation for one-step ahead forecasting. In the pseudo-out-of-sample strategy, the one-step-ahead forecast at time $t+\tau$ is estimated using the actual data through time $t$, then moving forward to time $t+\tau$ and repeating until all test data samples are covered in the recursive estimation. In this work, we used an increasing data window in the recursive forecast of samples in the test set.

See Supplementary Information’s materials and methods section for details on simulated data from ecosystem models and real data from mesocosm experiments.

## Results and Discussion

**Figure 3:**
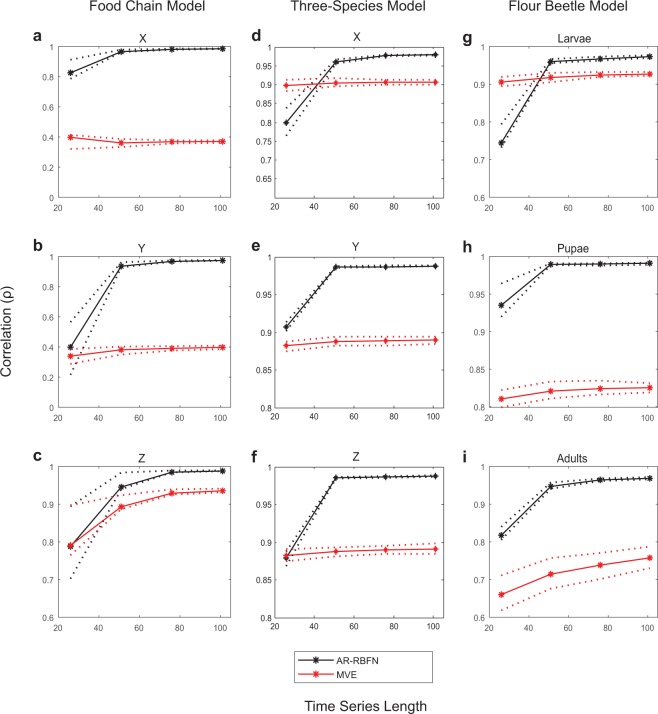
Comparison of forecast performance (correlation) of AR-RBFN and MVE using simulated ecological data with 10% added noise. **(a**–**c)** forecast skill (correlation between estimated forecast and one-step-ahead observation) versus time-series length of the data libraries for variables X, Y, and Z in three-species food chain model. **(d**–**f)** same as a to c but for the three-species coupled logistic model. **(g**–**i)** same as a to c but for variables larvae, pupae and adults in the flour beetle model. Solid lines show the averaged values for 100 randomly selected data libraries, and the dotted lines indicate the upper and lower quartiles.

To assess the performance of the AR-RBFN approach, we compare the forecast performance between the out-of-sample forecast estimates and the one-step-ahead observations using our proposed AR-RBFN autoregressive model with that of a model-free approach based on nearest neighbors, MVE, proposed by Ye *et al*.^20^. Figure 3 depicts the forecast skill (correlation) of the AR-RBFN and the MVE approaches for simulated ecological systems with 10% added noise for a three-species food chain^23^, a three-species coupled logistic and a three-stage flour beetle model^24^ (for details on MVE method see Supplementary Information). In almost all cases, AR-RBFN provides better forecast skills with higher correlations. As expected, the forecast performance improves as the length of time-series increases.

**Figure 4:**
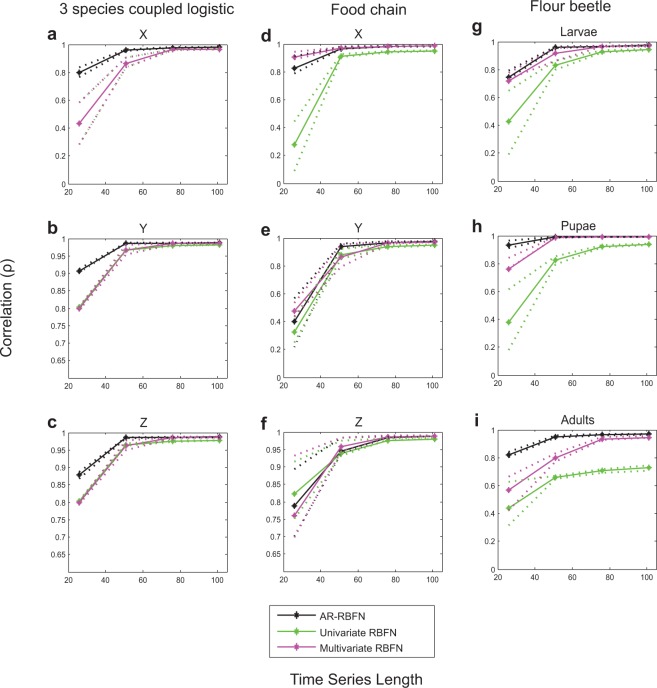
Forecast performance (correlation) vs. time-series length of libraries with 10% added noise. **(a**–**c)** Average correlation between predictions and observations for 100 randomly sampled libraries for variables $X$, $Y$, and $Z$ vs. length of the libraries in the 3 species coupled logistic model. **(d**–**f)** Same as a to c but for the food chain model. **(g**–**i)** Same as a to c but for the variables larvae, pupae and adults in the flour beetle model. The solid black curves are the average correlations for the attractor ranked RBFN approach for the top $k$ manifold reconstructions. The solid green curves are the average correlations for the univariate RBFN approach, and the solid pink curves are the average correlations using the multivariate model constructed by the variable combination with the best in-sample prediction skill in the RBFN autoregressive approach. The dotted lines are the upper and lower quartiles.

**Figure 5:**
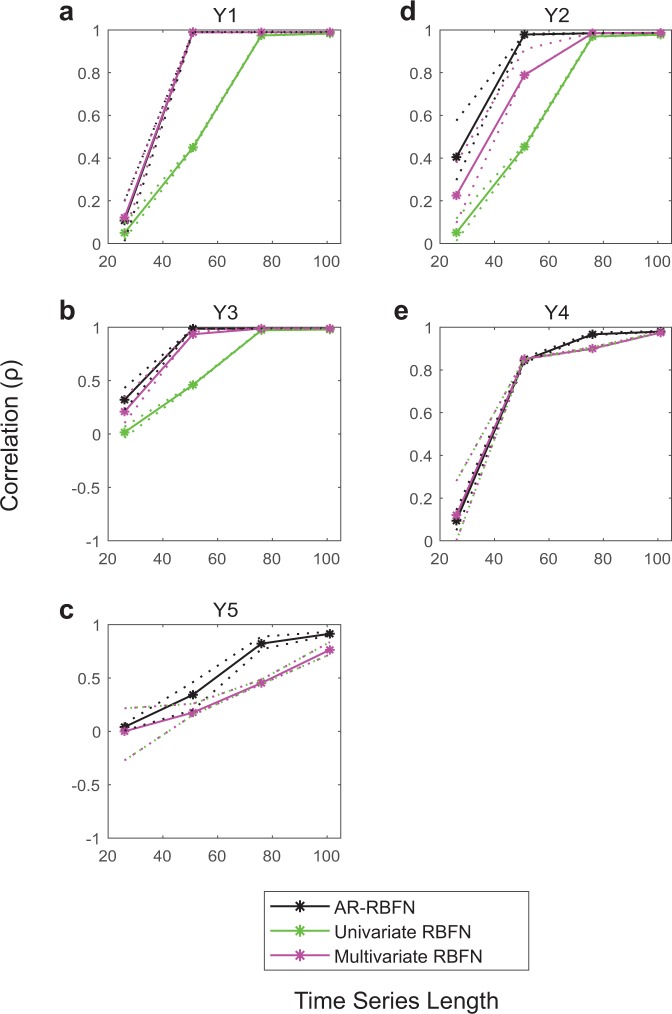
Forecast performance (correlation) vs. time-series length of libraries for the five-species model with 10% added noise. **(a**–**e)** Average correlation between predictions and observations for 100 randomly sampled libraries for variables ${Y}_{1}$, ${Y}_{2}$, ${Y}_{3}$, ${Y}_{4}$, ${Y}_{5}$ vs. length of the libraries. The solid black curves are the average correlations for the attractor ranked RBFN approach for the top $k$ manifold reconstructions. The solid green curves are the average correlations for the univariate RBFN approach, and the solid pink curves are the average correlations using the multivariate model constructed by the variable combination with the best in-sample prediction skill in the RBFN autoregressive approach. The dotted lines are the upper and lower quartiles. In figure panels c and e, the manifolds of the univariate and multivariate models with the best in-sample prediction coincide.

Furthermore, as shown in Figs. 4 and 5, AR-RBFN yields better forecast performance than that from a univariate radial basis function network and a radial basis function network using the multivariate model (constructed by the variable combination with the best in-sample prediction skill) for the three-species models and a five-species model^25^ (see Supplementary Information for details on univariate and multivariate RBFN as well as Figs. S2 and S3).

**Figure 6:**
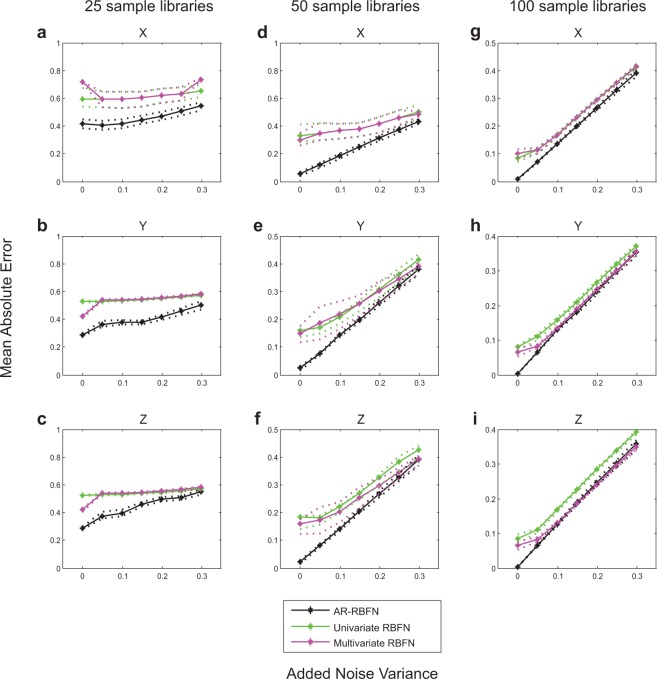
Forecast performance (mean absolute error) vs. noise for the 3 species coupled logistic model. **(a**–**c)** Average mean absolute error between predictions and observations for 100 randomly sampled libraries of length 25 for variables $X$, $Y$, and $Z$. **(d**–**f)** Same as a to c but for 100 randomly sampled libraries of length 50. **(g**–**i)** Same as a to c but for 100 randomly sampled libraries of length 100. The solid black curves are the average mean absolute errors for the attractor ranked RBFN approach for the top $k$ manifold reconstructions. The solid green curves are the average mean absolute errors for the univariate RBFN approach, and the solid pink curves are the average mean absolute errors using the multivariate model constructed by the variable combination with the best in-sample prediction skill in the RBFN autoregressive approach. The dotted lines are the upper and lower quartiles.

The strength of AR-RBFN in providing better forecast skill is especially obvious when time-series are short. As the length of time-series increases, the performances of the univariate and multivariate RBFN improve and reach that of AR-RBFN. To further study the modeling framework of AR-RBFN, we investigate the effect of observational noise in the time-series data. For instance, Fig. 6 shows the effect of observational noise in the three-species coupled logistic model for libraries of length 25, 50 and 100 samples (also see Supplementary Figs. S4 to S8). Unsurprisingly, our results indicate that as more noise is added to the data, the forecast error increases. As seen in Fig. 6, when dealing with noisy data, AR-RBFN provides better forecast than the univariate and multivariate RBFN-based autoregressive models.

**Figure 7:**
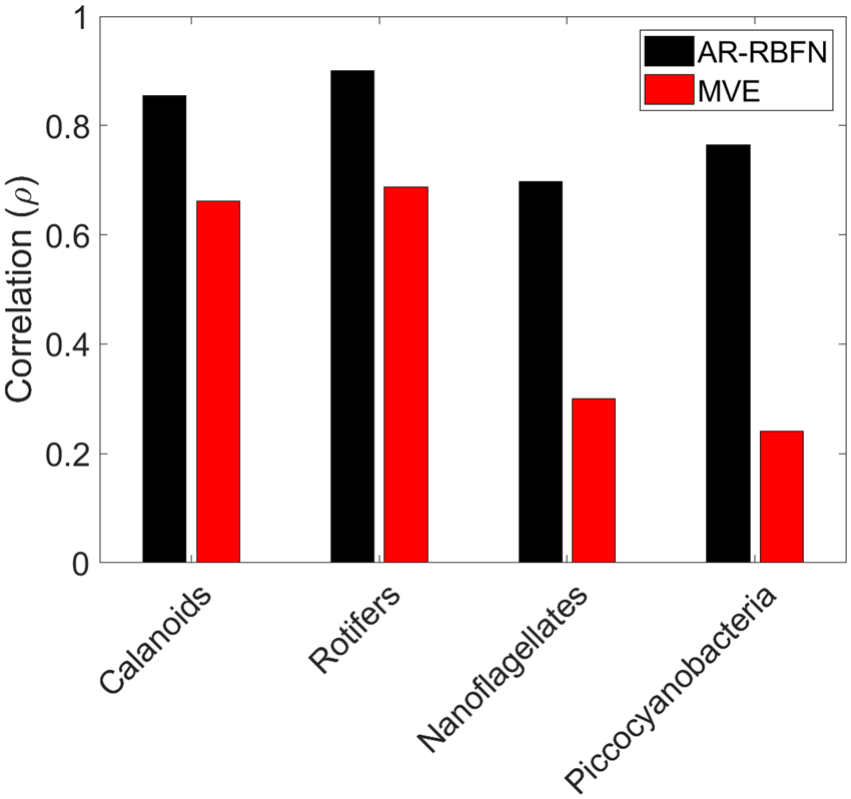
Comparison of forecast performance (correlation) of AR-RBFN and MVE for the long-term mesocosm experiment. Correlation between the predictions and observations for plankton communities of calanoids, rotifers, nanoflagellates and picocyanobacteria.

To further evaluate the forecast skill of AR-RBF on real world data, we extend this analysis to time-series data from a long-term mesocosm experiment on a four-species marine plankton community obtained from the Baltic Sea^26^. The mesocosm data consists of the plankton population of Nanoflagellates and Picocyanobacteria that fall prey to two predators, Rotifers and Calanoid Copepods. Coupling of predator-prey oscillations where preys have a causal effect on the predators exhibit chaotic patterns. Figure 7 shows the comparison of the forecast performances of AR-RBFN and MVE for the long-term plankton community data; for all four species, AR-RBFN outperforms MVE in forecasting. Using the MAE metric provides similar results when comparing MVE and AR-RBFN (see Supplementary Figs. S9 and S10).

We found that for attractor ranked radial basis function network (AR-RBFN), the best number of top $k$ reconstructions to incorporate into AR-RBFN is $k=N$, where $N$ is the number of variables in the interconnected dynamic system. If $k$ is too large, we will have too many hidden units in the hidden layer of the radial basis function network. Particularly in cases where the time-series is noisy, too many hidden units in the hidden layer of the neural network leads to overfitting of the training samples and poor generalization^27^. Here, we choose $\tau =1$ and $E=3$ for the ecosystem simulated data and mesocosm experiment data.

In this work, we designed a nonparametric forecasting algorithm based on state space reconstruction that can be generalized to any problem where the model structure is unknown. The incentive for developing this algorithm originates from the biological sciences, however it is possible to apply AR-RBFN to dynamic systems in the field of computer networks that arise from transmission control protocol (TCP)^28,29^, weather prediction^30^, or other applications.

We project the lagged observations from multiple components to state space trajectories and construct manifold attractors. The attractors’ prediction skill is then assessed, and the top manifolds are used for forecasting in a radial basis function network where a nonlinear function is estimated. This function maps the past events of the dynamic system into future values. This algorithm exploits the dimensionality of data in a nonparametric autoregressive framework, improving the ability to forecast the dynamical behavior of chaotic systems. Our method, AR-RBFN, computes the distance-weighted average of all points in the top $k$ manifolds (Fig. 2a). The Gaussian radial basis functions (activation functions) in the hidden layer produce higher values when the distance between the data points in the input manifolds and their corresponding prototypes (centers) are small; the activation values fall off exponentially as the distance between data points and prototypes increases^31^. As seen in Figs. 3 and 7, the estimated nonlinear function *f*(·) in AR-RBFN, which is a smooth map, produces better forecast performance than MVE which is a piece-wise constant function approximator.

When components of a complex dynamic system have cause-and-effect relationships with one another, relying on univariate information towards prediction of the system dynamics does not yield good predictions (Figs. 4, 5 and Supplementary Figs. S2 and S3). Our findings indicate that the information contained in pooled data enhances the prediction skill. AR-RBFN outperforms nonlinear univariate and multivariate autoregressive-based forecasting models since it exploits the pooled information contained in the top $k$ manifold reconstructions. The advantage of an attractor ranked prediction scheme is particularly evident when the time-series are short and noisy (Fig. 6 and Supplementary Figs. S4 to S8), a feature very common in biological and ecological data sets.

## Supplementary information

Supplementary Information File.

Dataset S1.

## Data Availability

Mesocosm experiment data is available in the appendix of Benica *et al*.^26^. Simulated data are available in Supplementary Table S1.
