# Supplementary material for: Attractor Ranked Radial Basis Function Network: A Nonparametric Forecasting Approach for Chaotic Dynamic Systems
Source: Sci Rep. 2020 Mar 2;10:3780. doi: 10.1038/s41598-020-60606-1 (PMC7052196; doi:10.1038/s41598-020-60606-1)
Supplement: Supplementary file 1 — Supplementary Information File. [file 41598_2020_60606_MOESM1_ESM.pdf]

Supplementary Information for

Attractor Ranked Radial Basis Function Network: A Nonparametric Forecasting Approach for Chaotic Dynamic Systems

Maryam Masnadi-Shirazi, Shankar Subramaniam*

*Correspondence to: shankar@ucsd.edu

This PDF file includes:

Supplementary Materials and Methods

Supplementary Figs. S1-S10

Caption for Supplementary Table S1

References

Other Supplementary Material for this manuscript includes the following:

Supplementary Table S1 (Excel file)

I. Materials and Methods

A. Simulated Data

The simulated data used in this work is generated from ecosystem simulations of a three-species food chain [1], a three-species coupled logistic model [2], a flour beetle model [3] and a five species model [4].

1. *Three-species food chain model*

The following differential equations model a chaotic three-species food chain of variables x, y, and z [1]:

$$dx/dt = x(1-x) - f_1(x)y$$

$$dy/dt = f_1(x)y - f_2(y)z - d_1 y$$

$$dz/dt = f_2(y)z - d_2 z$$

with

$$f_i(u) = a_i u/(1 + b_i u)$$

The parameter values used in the simulations are as follows: $a_1 = 2.5$, $a_2 = 0.1$, $b_1 = 3.2$, $b_2 = 2$, $d_1 = 0.2$, and $d_2 = 0.015$. The initial conditions used are $x_0 = 0.8$, $y_0 = 0.2$ and $z_0 = 8$.

2. *Three-species coupled logistic model*

The three interacting species x, y, and z are model through the following coupled logistic map as mentioned in Ye *et al*. (2016):

$$\begin{bmatrix} x(t+1) \\ y(t+1) \\ z(t+1) \end{bmatrix} = \begin{bmatrix} 3.6 \\ 3 \\ 3 \end{bmatrix} \circ \begin{bmatrix} x(t) \\ y(t) \\ z(t) \end{bmatrix} \circ \left(\begin{bmatrix} 1 \\ 1 \\ 1 \end{bmatrix} - \begin{bmatrix} 1 & 0.2 & 0.2 \\ 0.2 & 1 & -0.2 \\ 0.2 & -0.2 & 1 \end{bmatrix} \right)$$

where ° is the entry wise product. The initial conditions used in the simulations are $\begin{bmatrix} x(1) \\ y(1) \\ z(1) \end{bmatrix} = \begin{bmatrix} 0.2 \\ 0.2 \\ 0.2 \end{bmatrix}$.

3. *Flour beetle model*

The chaotic behavior of an insect population, *Tribolium Castaneum*, is modeled through the following equations for different life stages (larvae, pupae, and adults) of flour beetle suggested by Dennis *et al.* [3]:

$$L(t+1) = bA(t)\exp(-c_{e1}L(t) - c_{ea}A(t))$$

$$P(t+1) = L(t)(1-\mu_1)$$

$$A(t+1) = P(t)\exp\left(-c_{pa}A(t)\right) + A(t)(1-\mu_a)$$

with the following parameter values used in the simulations: $b = 10.67$, $\mu_1 = 0.1955$, $\mu_a = 0.96$, $c_{e1} = 0.01647$, $c_{ea} = 0.01313$, $c_{pa} = 0.35$. The initial values are $L(1) = 250$, $P(1) = 5$ and $A(1) = 100$.

4. *Five-species model*

The following equations identify a chaotic five-species competition model for variables Y_1, Y_2, Y_3, Y_4, and Y_5 suggested by Sugihara *et al.* [4]:

$$Y_1(t) = Y_1(t)[4 - 4\,Y_1(t) - 2\,Y_2(t) - 0.4\,Y_3(t)]$$

$$Y_2(t) = Y_2(t)[3.1 - 0.31\,Y_1(t) - 3.1\,Y_2(t) - 0.93\,Y_3(t)]$$

$$Y_3(t) = Y_3(t)[2.12 + 0.636\, Y_1(t) + 0.636\, Y_2(t) - 2.12\, Y_3(t)]$$

$$Y_4(t) = Y_4(t)[3.8 - 0.111\, Y_1(t) - 0.011\, Y_2(t) + 0.131\, Y_3(t) - 3.8\, Y_4(t)]$$

$$Y_5(t) = Y_5(t)[4.1 - 0.082\, Y_1(t) - 0.111\, Y_2(t) - 0.125\, Y_3(t) - 4.1\, Y_5(t)]$$

with the initial conditions $Y_1(1)$= $Y_5(1)$ =0.1, $Y_2(1)$=0.02, $Y_3(1) = Y_4(1)$=0.01.

B. Real Data

Mesocosm plankton community data

The data drawn from the mesocosm 8-year experiment on a plankton community isolated from the Baltic Sea has been shown to represent the dynamics of a chaotic system. We use the transformed data of the abundance of Rotifers, Calanoid Copepods, Picocyanobacteria and Nanoflagellates from the supplementary material of Benica *et al.* [5]. The data transformation in Benica *et al.* is done such that the raw data is interpolated by hermite cubic interpolation to obtain data with equidistant time intervals of 3.35 days, and then rescaled by a fourth-root transformation to suppress sharp peaks. The transformed data are of length 794 samples.

C. Manifold Reconstruction

As described in Ye *et al.* [2], the possible m number of 3-dimensional manifold reconstructions of combination of variables and their time lags of $0, \tau$ and 2τ is:

$$m = \binom{NL}{E} - \binom{N(L-1)}{E}$$

where N is the number of variables in the dynamic system, L is the number of possible lags for each variable, and E is the embedding dimension. The first term is the number of manifolds formed by choosing E of the NL possible coordinates, and the second term is subtracted to eliminate the number of invalid manifolds with E lagged coordinates. A valid manifold is one with at least one

unlagged coordinate. For example, the possible number of valid manifold reconstructions for a 3 and 4 variable system is 64, and 164 respectively. Unlike Ye *et al.* [2] that suggests $k = \sqrt{m}$, we found out that for multiview radial basis function network (MV-RBFN), the best number of top k reconstructions to incorporate into MV-RBFN is $k = N$, where N is the number of variables in the interconnected dynamic system. This is because for any N-variate system, if we let k be equal to $\sqrt{m}$ ($\sqrt{m} \geq N$) we will have too many hidden units in the hidden layer of the radial basis function network. Particularly in cases where the time series is noisy, too many hidden units in the hidden layer of the neural network leads to overfitting of the training samples and poor generalization [6]. In this work, we choose $\tau = 1$ and $E = 3$ for the ecosystem simulated data and mesocosm experiment data.

D. Multiview Embedding (MVE)

Multiview Embedding (MVE) is a forecasting algorithm that is based on Simplex Projection [7]. Simplex projection is a nearest neighbor forecasting technique that involves tracking the forward evolution of nearby points in an embedding, i.e., similar past events are used to forecast the future. Multiview Embedding (MVE) too involves reconstructing valid manifolds from combinations of variables and time lags [2]. In contrast to Simplex Projection where the forecast is based on weighted average of nearest neighbors, MVE examines the top k reconstructions and uses the single nearest neighbor from each to perform forecasting. For instance, the forecast of variable y is as follows:

$$\hat{y}_{t+1} = \frac{1}{k}\sum_{i=1}^{E+1} y_{nn^i(t)+1}$$

Where $nn^i(t)$ is the time index of the nearest neighbor in the ith attractor among the top k attractors. The number of possible reconstructions grows combinatorially with the number of

variables. Given l lags for each of the n variables the number of E-dimensional variable combinations:

$$m = \binom{nl}{E} - \binom{n(l-1)}{E}$$

In MVE prediction, the top $k = \sqrt{m}$ reconstructions, ranked by their in-sample forecast accuracy, are used.

Conventional simplex projection uses $E + 1$ nearest neighbors from a single attractor reconstruction, and each of these neighbors represents a unique historical state. Multiview embedding uses the single nearest neighbor from k attractor reconstructions.

E. Multivariate and Univariate RBFN

The difference between AR-RBFN and Multivariate and Univariate RBFN is in the manifold reconstructions that are fed as inputs to the radial basis function network. If we rank all the possible m manifolds based on their prediction skills in the in-sample portion of data, AR-RBFN takes the top k manifolds as the inputs whereas in the Multivariate RBFN model, the input of the RBFN is one single manifold with the best prediction skill among all m possible manifolds. The Univariate RBFN takes the single manifold that is reconstructed from lags of the variable we are forecasting, e.g., if we are to forecast the future of variable y, Univariate RBFN takes the manifold reconstructed from lags of y, namely $[y(t), y(t-\tau), y(t-2\tau)]$, as the input to the radial basis function network.

F. Computational Complexity

Ranking the manifold reconstructions in MVE algorithm involves using the simplex projection approach, which is based on nearest neighbors. The search for the nearest neighbors in all valid

manifold reconstructions in the simplex projection method leads to computational complexity of order $O(N.m.T)$, where N is the number of variables, m is the number of manifold reconstructions, and T is the number of samples in the time series. The computational complexity for finding the top k manifolds in the AR-RBFN approach is of order $O(m.p + N.m)$, where $m.p$ is related to time needed to find the p centers (prototypes) in each of the m manifold reconstructions and $N.m$ is related to the computational time required for building Gaussian radial basis functions (activation functions). Since $O(m.p + N.m) < O(N.m.T)$, AR-RBFN is of a lower computational complexity compared to MVE.

II. Supplementary Figures

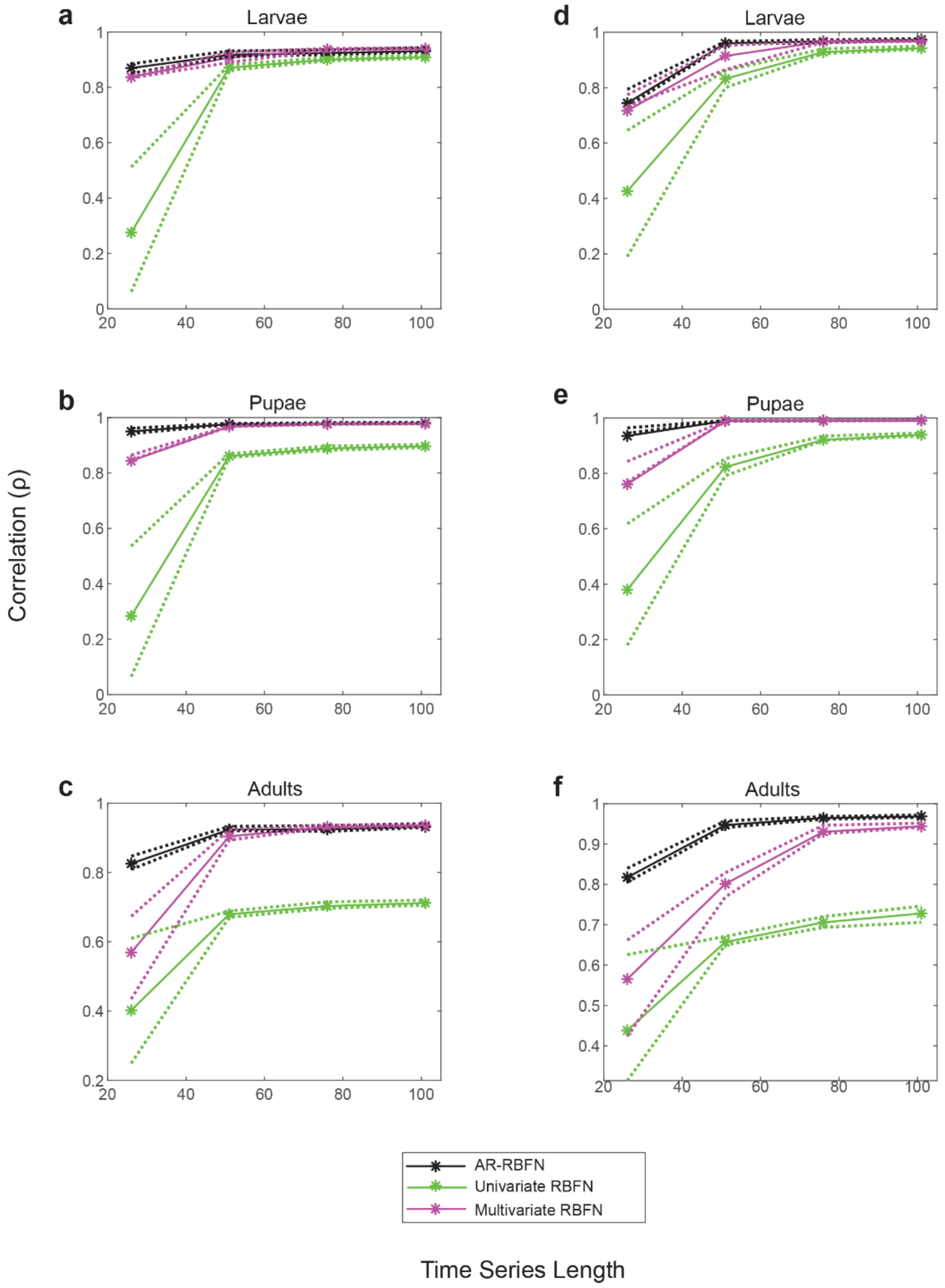

Fig. S1. Comparison of forecast performance of AR-RBFN in forecasting the intervals [2001, 2500] and [2501, 3000] for the Flour Beetle model. **(a-c)** average correlation between predictions and observations of larvae, pupae and adults in the [2001, 2500] time interval using 100 randomly sampled libraries. **(d-f)** average correlation between predictions and observations of larvae, pupae and adults in the [2500, 3000] time interval using 100 randomly sampled libraries.

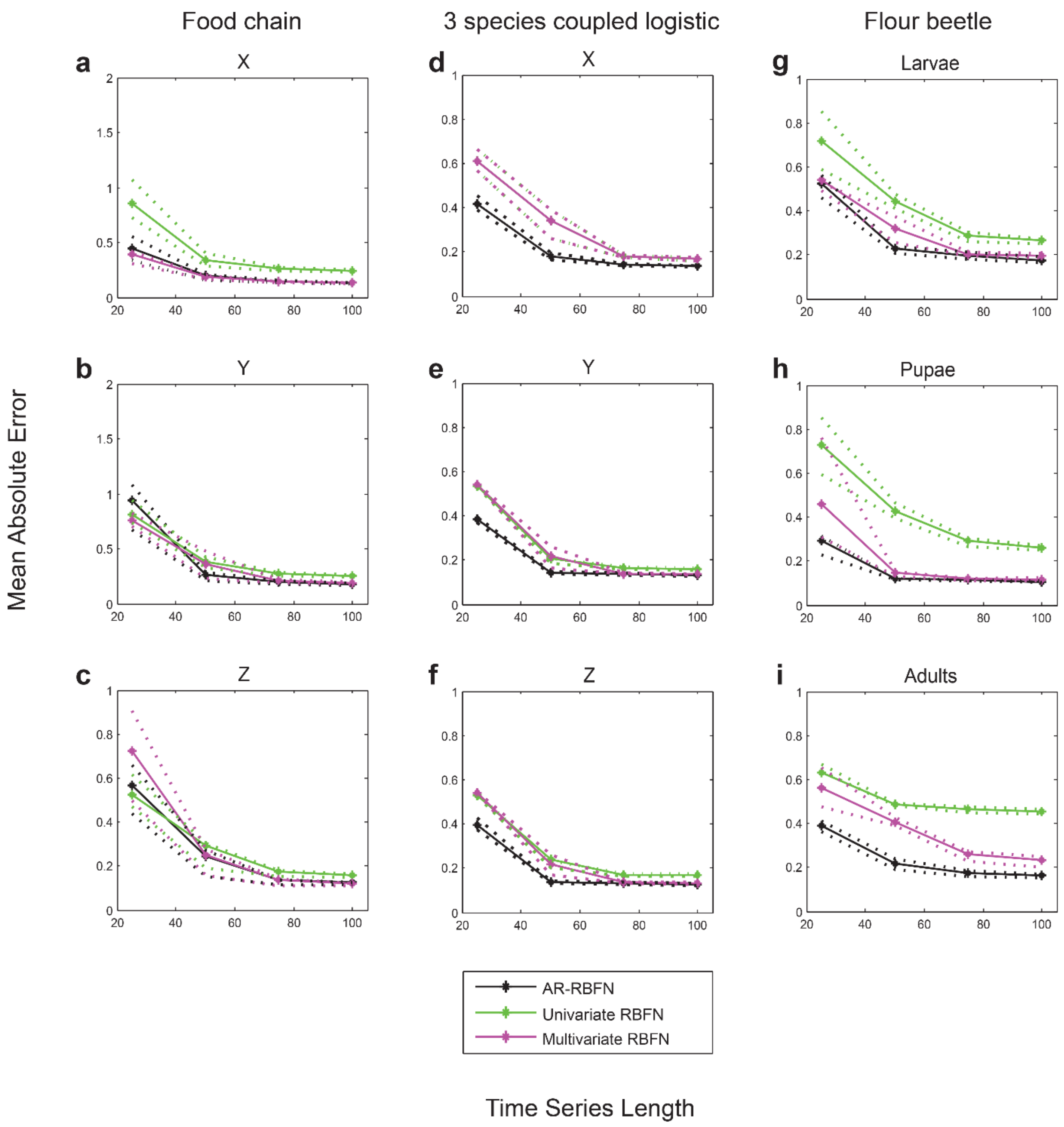

Fig. S2. Forecast performance (mean absolute error) vs. time series length of libraries with 10% added noise. **(a - c)** average mean absolute error between predictions and observations for 100 randomly sampled libraries for variables X, Y., and Z vs. length of the libraries in the food chain model. **(d - f)** same as a to c but for the 3 species coupled logistic model. **(g - i)** same as a to c but for the variables larvae, pupae and adults in the flour beetle model. The solid black curves are the average mean absolute errors for the attractor ranked RBFN approach for the top k manifold reconstructions. The solid green curves are the average mean absolute errors for the univariate RBFN approach, and the solid pink curves are the average mean absolute error using the multivariate model constructed by the variable combination with the best in-sample prediction skill in the RBFN autoregressive approach. The dotted lines are the upper and lower quartiles.

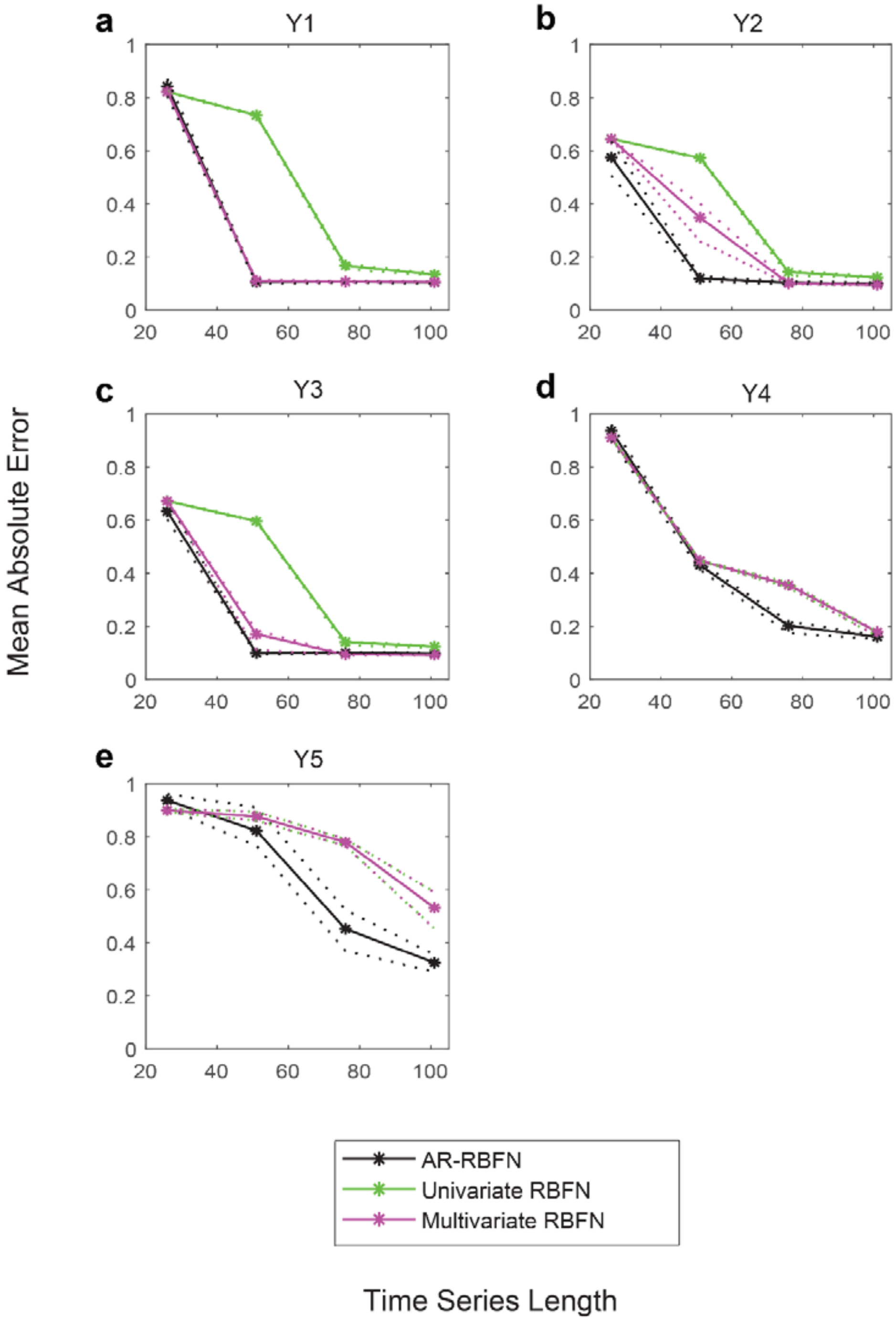

Fig. S3. Forecast performance (mean absolute error) vs. time series length of libraries for the five-species model with 10% added noise. **(a - e)** average mean absolute error between predictions and observations for 100 randomly sampled libraries for variables Y_1, Y_2, Y_3, Y_4, Y_5 vs. length of the libraries. The solid black curves are the average mean absolute error for the attractor ranked RBFN approach for the top k manifold reconstructions. The solid green curves are the average mean absolute error for the univariate RBFN approach, and the solid pink curves are the average mean absolute error using the multivariate model constructed by the variable combination with the best in-sample prediction skill in the RBFN autoregressive approach. The dotted lines are the upper and lower quartiles. In figure panels d and e, the manifolds of the univariate and the best single view coincide.

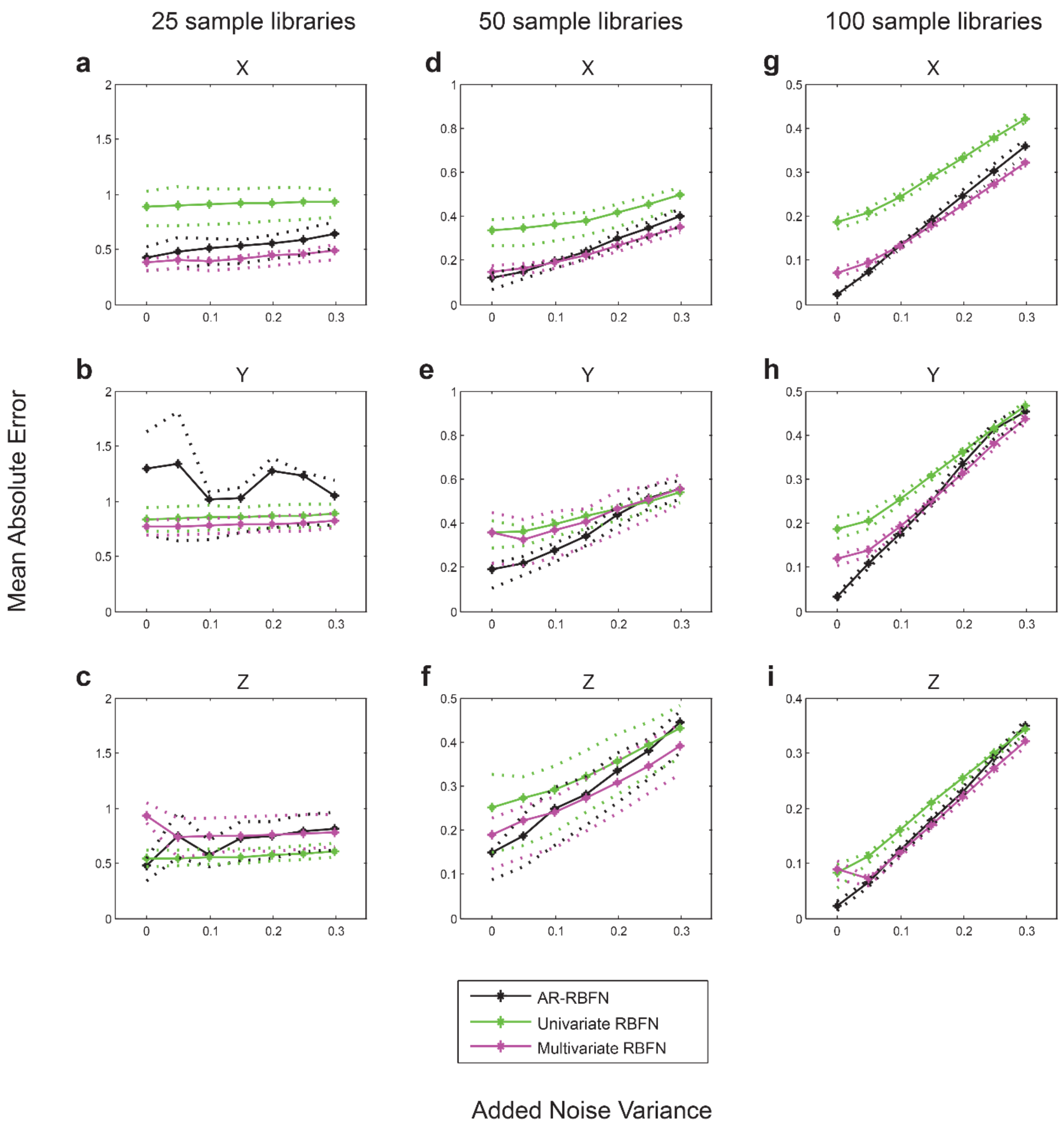

Fig. S4. Forecast performance (mean absolute error) vs. noise for the food chain model. **(a - c)** average mean absolute error between predictions and observations for 100 randomly sampled libraries of length 25 for variables X, Y, and Z. **(d - f)** same as a to c but for 100 randomly sampled libraries of length 50. **(g - i)** same as a to c but for 100 randomly sampled libraries of length 100. The solid black curves are the average mean absolute errors for the attractor ranked RBFN approach for the top k manifold reconstructions. The solid green curves are the average mean absolute errors for the univariate RBFN approach, and the solid pink curves are the average mean absolute error using the multivariate model constructed by the variable combination with the best in-sample prediction skill in the RBFN autoregressive approach. The dotted lines are the upper and lower quartiles.

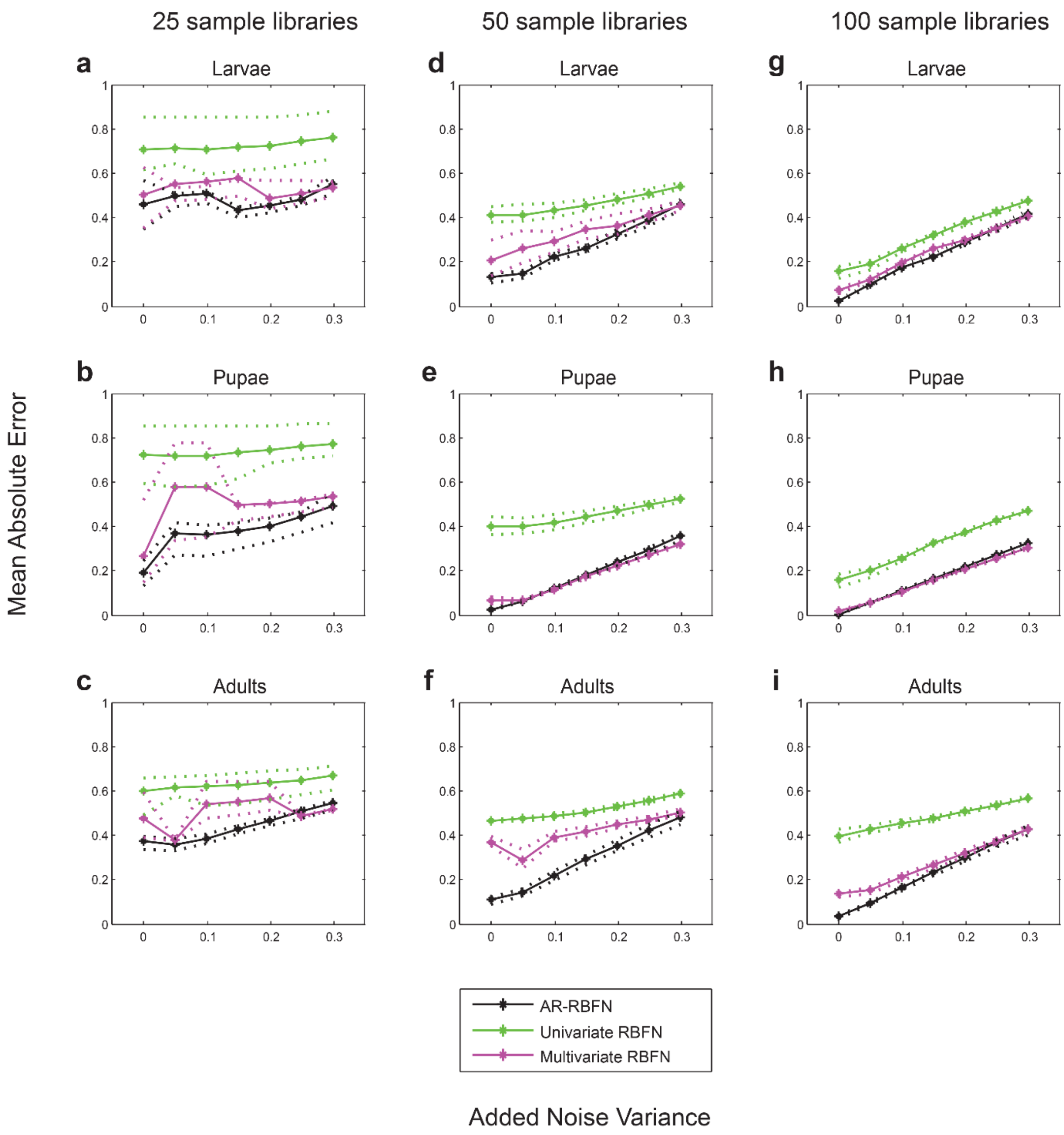

Fig. S5. Forecast performance (mean absolute error) vs. noise for the flour beetle model. **(a - c)** average mean absolute error between predictions and observations for 100 randomly sampled libraries of length 25 for variables larvae, pupae, and adults. **(d - f)** same as a to c but for 100 randomly sampled libraries of length 50. **(g - i)** same as a to c but for 100 randomly sampled libraries of length 100. The solid black curves are the average mean absolute errors for the attractor ranked RBFN approach for the top k manifold reconstructions. The solid green curves are the average mean absolute errors for the univariate RBFN approach, and the solid pink curves are the average mean absolute error using the using the multivariate model constructed by the variable combination with the best in-sample prediction skill in the RBFN autoregressive approach. The dotted lines are the upper and lower quartiles.

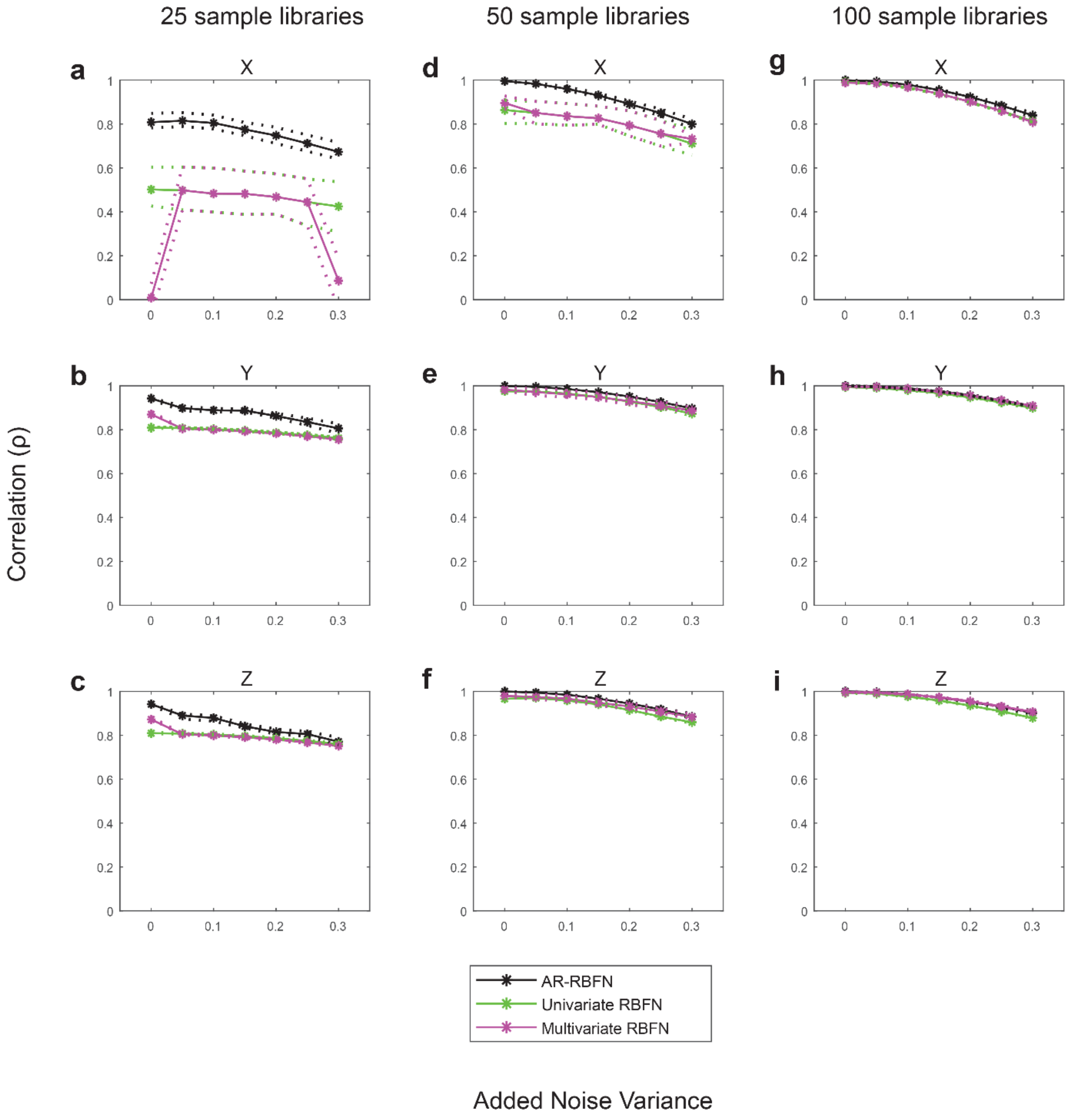

Fig. S6. Forecast performance (correlation) vs. noise for the 3 species coupled logistic model. **(a - c)** average correlation between predictions and observations for 100 randomly sampled libraries of length 25 for variables X, Y, and Z. **(d - f)** same as a to c but for 100 randomly sampled libraries of length 50. **(g - i)** same as a to c but for 100 randomly sampled libraries of length 100. The solid black curves are the average correlation for the attractor ranked RBFN approach for the top k manifold reconstructions. The solid green curves are the average correlation for the univariate RBFN approach, and the solid pink curves are the average correlation using the multivariate model constructed by the variable combination with the best in-sample prediction skill in the RBFN autoregressive approach. The dotted lines are the upper and lower quartiles.

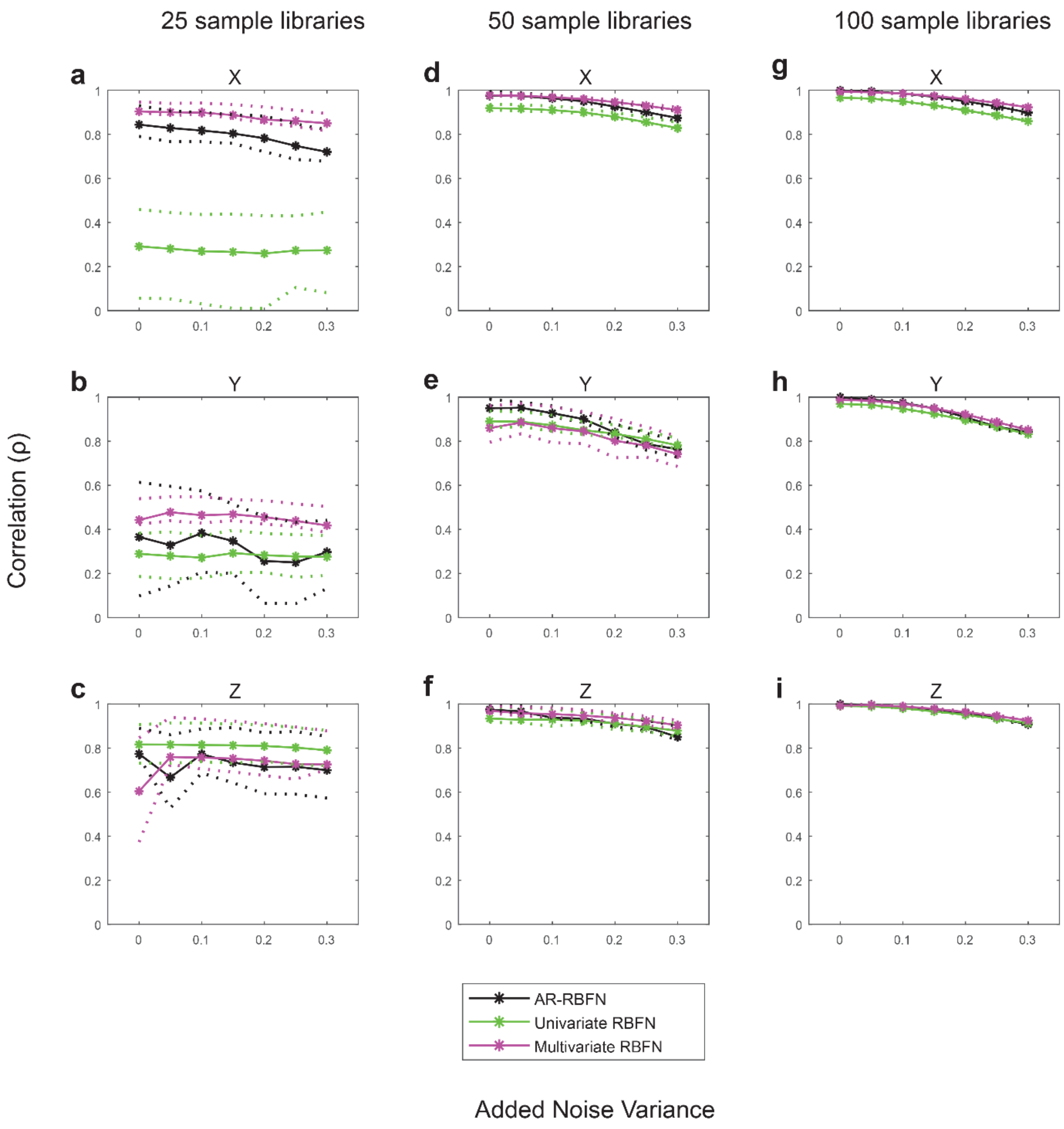

Fig. S7. Forecast performance (correlation) vs. noise for the food chain model. **(a - c)** average correlation between predictions and observations for 100 randomly sampled libraries of length 25 for variables X, Y, and Z. **(d - f)** same as a to c but for 100 randomly sampled libraries of length 50. **(g - i)** same as a to c but for 100 randomly sampled libraries of length 100. The solid black curves are the average correlation for the attractor ranked RBFN approach for the top k manifold reconstructions. The solid green curves are the average correlation for the univariate RBFN approach, and the solid pink curves are the average correlation using the multivariate model constructed by the variable combination with the best in-sample prediction skill in the RBFN autoregressive approach. The dotted lines are the upper and lower quartiles.

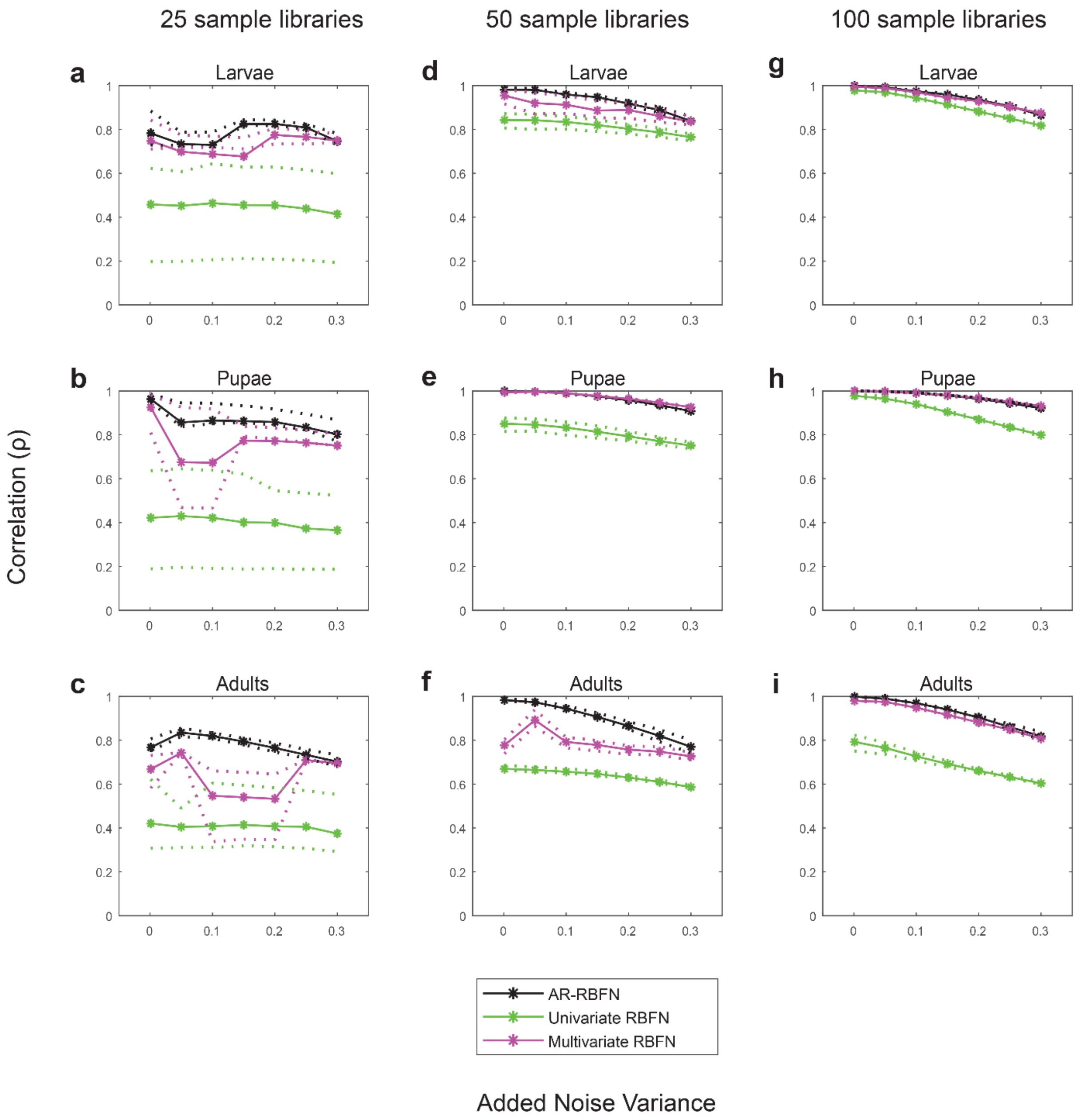

Fig. S8. Forecast performance (correlation) vs. noise for the flour beetle model. **(a - c)** average correlation between predictions and observations for 100 randomly sampled libraries of length 25 for larvae, pupae, and adults. **(d - f)** same as a to c but for 100 randomly sampled libraries of length 50. **(g - i)** same as a to c but for 100 randomly sampled libraries of length 100. The solid black curves are the average correlation for the attractor ranked RBFN approach for the top k manifold reconstructions. The solid green curves are the average correlation for the univariate RBFN approach, and the solid pink curves are the average correlation using the multivariate model constructed by the variable combination with the best in-sample prediction skill in the RBFN autoregressive approach. The dotted lines are the upper and lower quartiles.

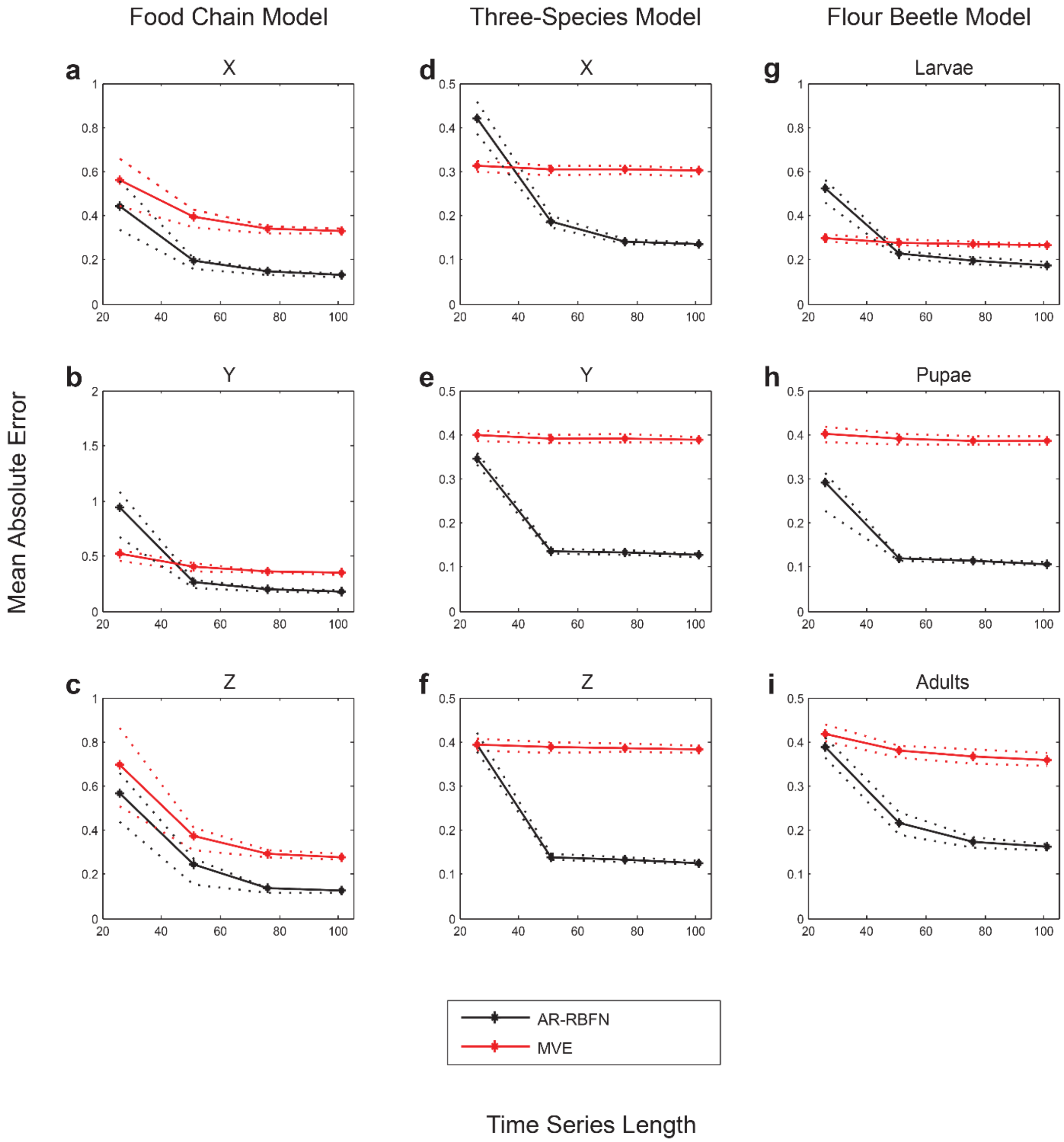

Fig. S9. Comparison of forecast performance (mean absolute error) for AR-RBFN and MVE in simulated ecological data with 10% added noise. **(a - c)** forecast skill (mean absolute error between estimated forecast and one-step-ahead observation) versus length of the libraries for variables X, Y, and Z in three-species food chain model. **(d - f)** same as a to c but for the three-species coupled logistic model. **(g - i)** same as a to c for the flour beetle model. Solid lines show the average values for 100 randomly selected libraries, and the dotted lines indicate the upper and lower quartiles.

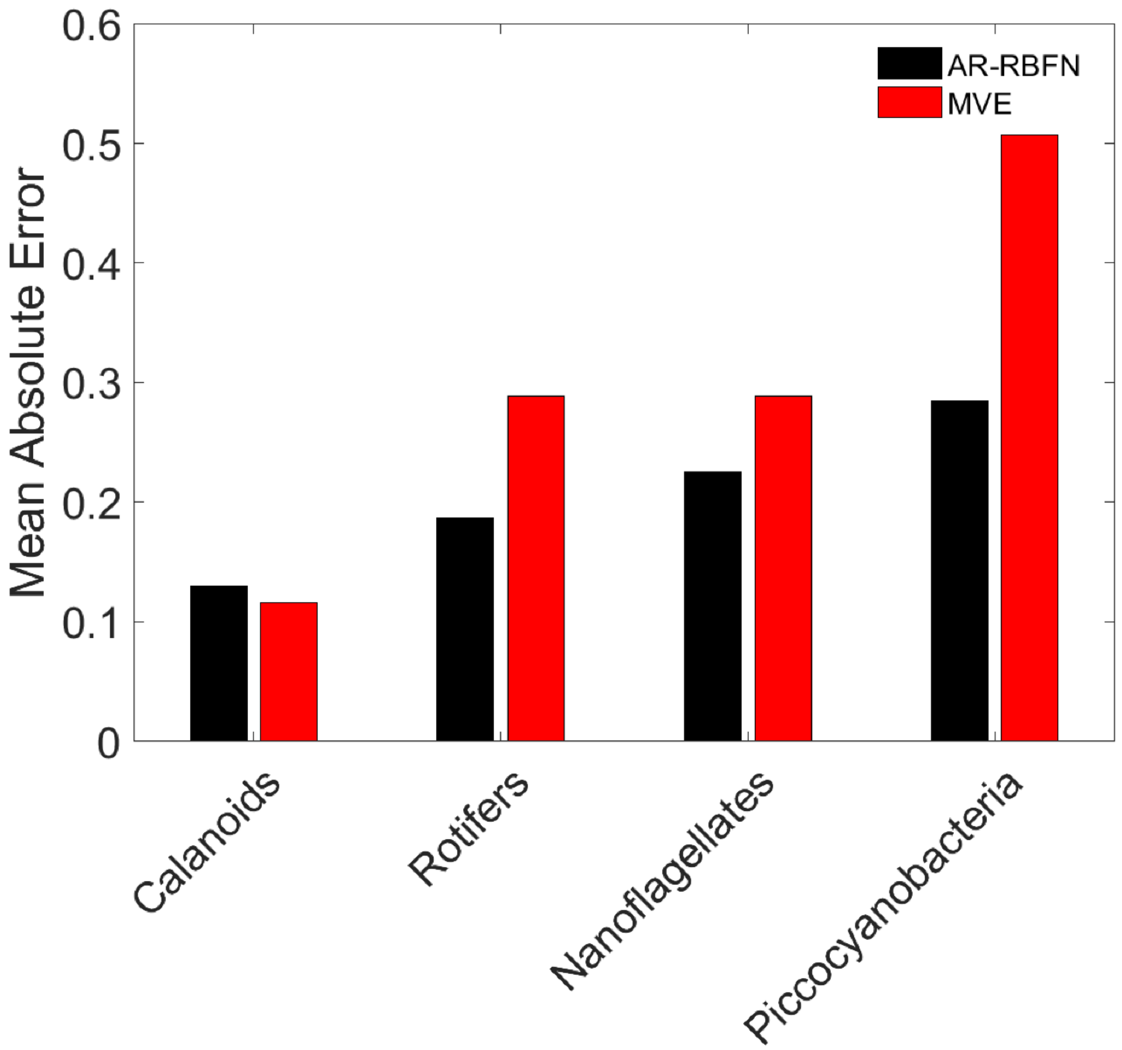

Fig. S10. Comparison of forecast performance (mean absolute error) of AR-RBFN and MVE for the long-term mesocosm experiment. Mean absolute error between the predictions and observations for plankton communities of calanoids, rotifers, nanoflagellates and picocyanobacteria.

III. Caption for Supplementary Table S1

This excel file contains the data generated from simulated ecosystem models for the three-species food chain model, the three-species coupled logistic model, the three-stage flour beetle model, and the five species model.

IV. References

1 Hastings, A. & Powell, T. Chaos in a three-species food chain. *Ecology* **72**, 896-903 (1991).
2 Ye, H. & Sugihara, G. Information leverage in interconnected ecosystems: Overcoming the curse of dimensionality. *Science* **353**, 922-925 (2016).
3 Dennis, B., Desharnais, R. A., Cushing, J., Henson, S. M. & Costantino, R. Estimating chaos and complex dynamics in an insect population. *Ecological Monographs* **71**, 277-303 (2001).
4 Sugihara, G. *et al.* Detecting causality in complex ecosystems. *science* **338**, 496-500 (2012).
5 Benincà, E., Jöhnk, K. D., Heerkloss, R. & Huisman, J. Coupled predator–prey oscillations in a chaotic food web. *Ecology letters* **12**, 1367-1378 (2009).
6 Leung, H., Lo, T. & Wang, S. Prediction of noisy chaotic time series using an optimal radial basis function neural network. *IEEE Transactions on Neural Networks* **12**, 1163-1172 (2001).
7 Sugihara, G. & May, R. M. Nonlinear forecasting as a way of distinguishing chaos from measurement error in time series. *Nature* **344**, 734 (1990).
